# Child functioning and disability in children living with human immunodeficiency virus in a semi-rural healthcare setting in South Africa

**DOI:** 10.4102/phcfm.v12i1.2259

**Published:** 2020-07-30

**Authors:** Stacy T. Maddocks, Lindokhule Mthethwa, Verusia Chetty

**Affiliations:** 1 Department of Physiotherapy, Faculty of Health Sciences, University of KwaZulu-Natal, Durban, South Africa; 2 Department of Physiotherapy, St Mary’s Hospital, Mariannhill, Durban, South Africa

**Keywords:** HIV, children, function, disability, impairments, healthcare, South Africa

## Abstract

**Background:**

Children living with HIV (CLHIV) often experience HIV-related impairment and disability.

**Aim:**

The study sought to understand the level of child functioning and access to rehabilitative care in the context of South African healthcare in order to inform an integrated rehabilitative framework.

**Setting:**

District level semi-rural healthcare facility in KwaZulu-Natal.

**Methods:**

The Washington Group/United Nations International Children’s Emergency Fund Module on Child Functioning, was administered to carers of CLHIV aged between 5 and 10 years, and accessing care at the study setting.

**Results:**

Forty-four caregivers of children receiving treatment from June 2018 to March 2019, at the facility, participated. Four (9.1%) children had difficulty with seeing, 13 (29.5%) children had difficulty with hearing and 10 (22.7%) children had difficulty with walking. In the cognitive and behavioural domains, 17 (38.6%) children reported difficulties in communication and concentration, with 16 (36.4%) children experiencing difficulties in learning and remembering. Difficulties reported in accepting change and controlling behaviour were both experienced by 23 (52.3%) children. Although many children experiencing impairments were referred for rehabilitation, many caregivers did not follow-up after the initial assessment, because of financial constraints, lack of time and transport restrictions.

**Conclusion:**

Functional difficulties were frequently experienced by children living with HIV. Disability screening would be beneficial at various points of care to promote early identification and timely referral to healthcare professionals. Decentralising rehabilitative care to homes and communities could offer a solution to some of the reported barriers to accessing care.

## Introduction

A significant number of children experience disabilities as a result of living with human immunodeficiency virus (HIV), including those on antiretroviral therapy (ART). Globally, 1.8 million children are living with HIV, of which approximately 280 000 children under the age of 14 are living in South Africa.^1^ Antiretroviral therapy adds years to the lives of children, but the virus leads to chronic and episodic, physical and cognitive manifestations, which influence their lives and everyday social participation.^2^ Physical impairments manifest in musculoskeletal conditions,^3,4^ visual^2,5,6^ and auditory impairments;^7,8^ as well as in speech and language deficits.^9,10^ Neurological and cognitive impairments also influence the development of children living with HIV (CLHIV) bearing consequences for achievement at school.^2,11,12,13^ These impairments can result in functional and participatory limitations, further compounded by the effects of contextual barriers resulting in disabilities. Although scholars and health professionals have recently adopted a biopsychosocial framework^14^ to understand disability, less is understood about HIV-related disability. Human immunodeficiency virus-related disability is understood by considering the life-related consequences of the disease as through the international classification of functioning, disability and health framework of impairments in body structure or function, limitations to activity as well as participation and environmental restrictions.^14^ Human immunodeficiency virus-related disability is thought to result from the neurotoxic characteristics of the virus, from the side effects of antiretrovirals and from opportunistic infections such as meningitis and otitis media.^2,10^ Additionally, the influence of the child’s social context on disability outcomes is acknowledged.^14^ Whilst some sub-Saharan research has investigated the prevalence and types of disabilities in CLHIV,^15^ this research interest is still relatively novel in resource-poor settings such as in South Africa where HIV is such a major health concern.

Furthermore, current paediatric HIV care in South Africa is not linked with routine disability screening or rehabilitation.^15^ Rehabilitation is fundamental in addressing not merely the impairments associated with HIV but also the accommodation of children in communities and society as a whole. However, access to rehabilitation in resource-poor settings is often left to the discretion and referral practices of the attending physician. This study is part of an umbrella project that aims to assess the feasibility (acceptability, practicality and preliminary efficacy) of an integrated model of rehabilitation and paediatric HIV care in order to improve the identification of and interventions for disability in CLHIV.^16^ The purpose of this study is to determine the level of child functioning and the prevalence and types of disabilities in CLHIV, aged between 5 and 10 years, in a resource-poor setting and to also investigate the access to rehabilitation. This study is crucial in the advocation for early interventions for children and rehabilitation to strengthen paediatric HIV care in the region.

## Methods

A cross-sectional survey to investigate the prevalence of disabilities was conducted with caregivers of CLHIV and accessing care from a public hospital in KwaZulu-Natal, South Africa. The study setting is described as a semi-rural community, based on its geographical location as an out-of-town suburb on the periphery of Durban. The total population of 58 caregivers of CLHIV aged between 5 and 10 years, on ART and on the hospital medical records from June 2018 to March 2019 were all invited to participate in the survey during their routine clinic visits. Only primary caregivers of CLHIV were included in the survey interviews in an attempt to obtain the most reliable information.

### Measurements

The questionnaire consisted of a socio-demographic section, a general medical history section and the Washington Group/United Nations International Children’s Emergency Fund (UNICEF) Module on Child Functioning. The Washington Group/UNICEF Module on Child Functioning covers children aged between 2 and 17 years and assesses functional difficulties in different domains, including hearing, vision, communication or comprehension, learning, mobility and emotions. The tool uses a rating scale for degrees of functionality in each domain^17^ and allows for the use of different ‘cut-offs’ for disability, namely ‘some difficulty’, ‘a lot of difficulty’ and ‘cannot do at all’. For the purpose of this study, a child was considered to be experiencing disability at the level of ‘some difficulty’ with carrying out functional activities.^18^ The tool has shown good diagnostic accuracy^19^ in international research, and studies suggest using the cut off ‘some difficulty’ to identify children at risk of disability and then refer to a medical practitioner for an objective assessment.^18,19^

### Procedures

Researchers conducted the survey during the children’s routine HIV follow-up clinic visits to the hospital facility or during special visits planned by the research team at nearby clinics between June 2018 and March 2019. The survey was conducted in English or IsiZulu (as preferred by the caregivers). The survey was translated into isiZulu by an isiZulu language specialist with backward translation thereafter to validate the translation. Survey results were captured on a tablet and uploaded onto a Microsoft Excel spreadsheet. Participants were coded to ensure anonymity. Medical charts were also used to extract relevant medical information such as co-morbidities and care management approaches.

### Data analysis

Data captured from the questionnaires were entered into Microsoft Excel and analysed using the Statistical Package for the Social Science (SPSS), version 25. Descriptive statistics were used to assess outliers and identify missing values. Categorical variables were presented in tables.

### Ethical consideration

Ethical approval to conduct the study was obtained from the University of KwaZulu-Natal’s Biomedical Research Ethics Committee (BFC386/17). Approval was granted by the relevant authorities and informed consent was obtained from all caregivers, who were assured that their participation would be strictly voluntary, prior to the commencement of the study.

## Results

Forty-five caregivers agreed to be interviewed. The mean age of the children was 7.77 years (standard deviation [s.d.] = 1.78). One participant had to be excluded because of missing data, resulting in 44 participants who were included in the final analysis. Participant characteristics are shown in Table 1.

**TABLE 1:** Participant and children living with human immunodeficiency virus characteristics (*n* = 44).

Variables	%	Frequency
**Child’s gender**		
Male	47.7	21
Female	52.2	23
**Schooling**		
Attending school	93.2	41
Age appropriate grade	59.1	26
Failed a grade	29.5	13
**Primary caregiver**		
Mother	56.8	25
Father	4.5	2
Adult relative	31.8	14
Non-relative adult	6.8	3
**Orphan status**		
Orphaned	34.1	15
**Mother on ARTs during pregnancy**		
Yes	15.9	7
Unknown	9.3	4
No	75	33
**Previous reported co-morbidities**		
Clinical encephalopathy	9.1	4
Developmental delay	22.7	10
Epilepsy	11.4	5
Meningitis	4.5	2
Otitis media	11.4	5
Pulmonary tuberculosis	38.6	17
**ART adherence**		
Good reported ART adherence	84.1	37

ART, antiretroviral therapy.

Mothers of the children – the majority of whom were not receiving ART during pregnancy – made up more than half of the primary caregivers. The other caregivers included fathers, grandmothers and aunts; and a small percentage of them were adults who were not biologically related to the family. A number of the children had received diagnoses of developmental delay or epilepsy from the doctors and/or had been previously infected by opportunistic pathogens leading to co-morbidities. A majority of the children attended school; however, 41% of them were not in the appropriate grade for their age because of caregiver-reported learning difficulties. The level of child functioning – as reported by the caregiver – is attached (Appendix 1) with interpretation reflected in Table 2.

**TABLE 2:** Tabulation of prevalence of functional difficulty among children aged 5–10 years.^17,18^

Functional domains	Functional difficulty if the following is true	Prevalence of functional difficulty in this study population (*n* = 44)	
		*n*	%
Seeing	If CF2 = 3 OR CF2 = 4 OR If CF3 = 3 OR CF3 = 4 Added CF2 = 2 and CF3 = 2^17^	4	9.1
Hearing	If CF5 = 3 OR CF5 = 4 OR If CF6 = 3 OR CF6 = 4 Added CF5 = 2 and CF6 = 2^17^	13	29.5
Walking	If CF8 = 3 OR CF8 = 4) OR (CF9 = 3 OR CF9 = 4) OR If CF12 = 3 OR CF12 = 4) OR (CF13 = 3 OR CF13 = 4) Added CF8 = 2, CF9 = 2, CF12 = 2 and CF13 = 2^17^	10	22.7
Self-care	CF14 = 3 OR CF14 = 4 Added CF14 = 2^17^	10	22.7
Communication (being understood inside or outside the household)	CF15 = 3 OR CF15 = 4 OR CF16 = 3 OR CF16 = 4 Added CF15 = 2 and CF16 = 2^17^	17	38.6
Learning	CF17 = 3 OR CF17 = 4 Added CF17 = 2^17^	16	36.4
Remembering	CF18 = 3 OR CF18 = 4 Added CF18 = 2^17^	16	36.4
Concentrating	CF19 = 3 OR CF19 = 4 Added CF19 = 2^17^	17	38.6
Accepting change	CF20 = 3 OR CF20 = 4 Added CF20=2^17^	23	52.3
Controlling behaviour	CF21 = 3 OR CF21 = 4 Added CF21 = 2^17^	23	52.3
Making friends	CF22 = 3 OR CF22 = 4 Added CF22 = 2	12	27.3
Anxiety	CF23 = 1	2	4.5
Depression	CF24 = 1	1	2.3

CF, child functioning.

These results reflect the caregiver-reported levels of child functioning^17^ and are tabulated in accordance with Loeb et al.^18^ For the purpose of this study, the cut-off for disability was considered as ‘some difficulty’ in functioning as reported by caregivers (the authors added ‘some difficulties’ as reported by participants to the composite scoring in Table 3). Disability can be conceptualised on a continuum from minor difficulties in functioning to major impacts on a person’s life. Therefore, the categories are designed to reflect this continuum, with cut-offs that can determine disability for the population under investigation.^18^

**TABLE 3:** Referral to rehabilitation professionals.

Support (allied health services)	Total number of participants (*n* = 44) referred to support services	Number of children referred (*n* = 22) who accessed support services
Optometry	4	4
Audiology	5	5
Physiotherapy	8	5
Occupational therapy	2	2
Speech therapy	3	2

**Total**	**22**	**18**

The results reflect that only four caregivers reported ‘some’ or ‘a lot’ of difficulty in the *seeing* domain, with 13 reporting ‘some’ or ‘a lot’ of difficulty within the *hearing* domain (29.5%). Ten caregivers reported ‘some’ or ‘a lot’ of difficulty in the *walking* domain, with two caregivers in the *self-care* domain reflecting that the CLHIV ‘cannot do at all’. In the cognitive and behavioural domains, findings reflect that 38.6% of the participants reported difficulties in *communication* and *concentration*, with a number of caregivers (36.4%) reporting difficulties in *learning* and *remembering*. Difficulties were reported in *accepting change* and *controlling behaviour* by 52.3% of caregivers. There were minor difficulties in *making friends*, and/or *anxiety* and *depression* reported by caregivers. The referral of CLHIV to rehabilitation professionals at a point of care whilst receiving medical management for HIV at the healthcare facility is reflected in Table 3.

Most CLHIV who were referred to rehabilitation professionals accessed care for an initial assessment on the same date of referral; however, a few of them did not follow up with their referral for rehabilitation. Seventeen caregivers did not return for follow-up treatment after the initial assessment. The reasons provided by caregivers for non-attendance at follow-up treatment included the distance to travel from their home to the centralised healthcare facility. Caregivers also believed that it was time consuming and transport costs were not affordable. Some caregivers of the CLHIV made personal requests for referral to other allied healthcare professionals such as an HIV counsellor (*n* = 1), dentists (*n* = 12) and psychologists (*n* = 4).

## Discussion

The study aimed to determine the level of function and prevalence of disability among a cohort of CLHIV between the ages of 5 and 10 years old who access care at a public healthcare facility in KwaZulu-Natal, South Africa. The Washington Group/UNICEF Module on Child Functioning was used to interview caregivers of CLHIV in order to screen for difficulties in functioning.^18,19^ The child functioning module measures difficulties with functional activities and grades them according to the level of difficulty experienced. To identify even the mildest difficulty in functioning, we used the cut-off ‘some difficulty’ with functioning to indicate disability^18^ as an initial screen for onward referral and objective assessment.

Although the Washington Group/UNICEF Module on Child Functioning used was designed for population-based comparisons of disability data,^18^ the tool yields results that may assist in informing the integration of rehabilitation into the current healthcare system for CLHIV. Functional difficulties were identified across physical, cognitive and behavioural domains in CLHIV. Whilst disability prevalence studies in sub-Saharan Africa^2,10,15^ have highlighted impairments frequently experienced by CLHIV using tools such as the ten question screen for disability (TQSD); this tool does not report on actual levels of child functioning. It was hoped that the identification of even the mildest level of functional difficulties using this tool could lead to early appropriate referral to medical professionals for objective assessment and timeous intervention.

Overall, the percentage of difficulties in cognitive functioning domains like remembering, learning and concentrating was reported more frequently than that of seeing, hearing, walking and self-care difficulties. The results were unsurprising as impaired cognitive functioning among CLHIV is well documented in the literature^20^ but in a context like South African healthcare where the burden of HIV is among the poorest of the poor, cognitive functioning difficulties become a significant concern for school access, success and eventually future livelihoods.^21,22^ Communication difficulties were also frequently reported in this study and could have resulted from neurocognitive impairment, neuromotor impairment or even the lack of verbal stimulation. Poor language development outcomes in CLHIV may be a result of a hearing impairment caused by HIV-related meningitis or recurrent otitis media infection.^7,8,23^ Furthermore, poor language development outcomes may be directly related to poor motor functioning, where overall muscle weakness affects oral-motor functioning.^10^ As this study reports an increased frequency of hearing difficulties for CLHIV, these results may have influenced the functioning domain scores of communication and learning. The interplay between hearing difficulties, language development and learning which may influence academic success^7^ highlights the imperative for early identification of functional difficulties and referral for timely, appropriate management. Perhaps these findings could explain the high percentage of children who were not in the appropriate school grade for their age. Apart from academic performance, just over half the sample were described to have experienced behavioural and mental health difficulties, all of which may contribute to much larger social issues of participation at school, like forming friendships.

Further to the pathogenic effects of HIV on neurocognitive and neuromotor outcomes in children, researchers agree that often impediments in the development and functioning of CLHIV is beyond pathology and strongly associated with the socio-economic status and quality of the household environment, especially in African settings.^24,25^ In this study, although most of the caregivers of CLHIV were their biological mothers, one-third of the children were orphaned (had lost one or both parents). An orphan status has contextual relevance for the development and functioning of CLHIV, particularly in resource-poor settings like the study setting where children may be required to forfeit education to care for the remaining parent or take up employment to supplement the household income. This is especially relevant in South Africa where the intersectionality between HIV and poverty, poverty and disability and HIV and disability is so apparent.^24^

Many studies in sub-Saharan Africa and those around the world^26,27,28^ have acknowledged the biopsychosocial mechanisms such as socio-economic factors that need to be addressed to improve the care offered to CLHIV. The findings of this study accentuated the multi-systemic manifestations of HIV in children^29,30,31^ and highlight the need for a multidisciplinary team approach to integrated healthcare management.

Most of the CLHIV who were referred for rehabilitation accessed those services for the initial consult only. Caregivers imputed their lack of follow-up to a lack of transport, financial constraints and the distance from their homes to the centralised public healthcare facility. The barriers concur with those identified in studies in similar contexts.^2,15,32,33^ Researchers have highlighted that although HIV-related disability is frequently experienced by children, there is an unmet need for rehabilitation services in the African context.^2,15^ Furthermore, in this study, participants flagged the need for other very necessary services, such as psychology and dentistry, which were not available at the study setting. The results specifically point to a need for referral pathways to audiologists, speech and language pathologists, occupational therapists, psychologists and physiotherapists in from the physicians and nurses attending to CLHIV.

The researchers understand that advocating for the identification of functioning difficulties or disability and subsequent referral may impose an additional load on already strained health systems. Therefore, more innovative healthcare delivery systems in resource-poor settings where people are experiencing a double burden of health and financial difficulties is needed.^34,35^ A task-shifting approach (training of lay personnel to provide necessary community-based or home-based healthcare services) in communities where health systems are overburdened has been widely regarded as an acceptable mechanism to provide care, health promotion and health education in areas where access is challenged.^33,36^ This approach to rehabilitation for adults living with HIV has been piloted with great success in the region of the study setting.^33^ The challenges of this approach in our context may be the cost implications but studies show that training the lay personnel to screen and execute simple home-based treatment strategies across the healthcare disciplines are more cost effective than employing trained professionals.^36^ However, feasibility studies that assess the cost of task-shifting healthcare services for CLHIV versus the benefits to individuals and centralised healthcare facilities may need further exploration. To our knowledge this is the first study to determine the level of functioning and disabilities in CLHIV aged 5 to 10 years using the Washington Group/UNICEF Module on Child Functioning in South Africa. It is important to note that caregiver reports may provide varying degrees of functional difficulty, based on their own perceptions, which may differ from the child’s perception of functioning or an objective assessment of levels of function.^19^

## Conclusion

The study highlights that functional difficulties are frequently experienced by CLHIV, which could potentially hinder their participation in ordinary activities such as school and play. These findings prompt the need for routine, deliberate disability-screening practices for CLHIV in resource-poor settings as part of their standard HIV care. Disability screening in CLHIV at various points of care, including primary healthcare sites, may be a step in the right direction for early identification and referral to rehabilitation. Community-based rehabilitation programmes that include the integration of all medical and paramedical support services, as well as mental health services, into standard paediatric HIV care are a potential pathway to upscale HIV paediatric care in order to promote the participation of children in their communities.

## Limitations

The Washington Group/UNICEF Module on Child Functioning is a relatively new tool used for national surveys and has not been validated in the South African community context. This was a single site study with a small population and no control group. Although the study provides a glimpse into the functional challenges encountered by CLHIV in a resource-poor setting in KwaZulu-Natal, further investigation into the factors associated with each impairment is crucial.

The researchers acknowledge that these are caregiver-reported functional difficulties, rather than objectively measured functional difficulties. Whilst the objective assessment is a critical next step, this does not diminish the importance of screening for functional difficulties in CLHIV at various points of healthcare.

The International Classification of Functioning, Disability and Health (ICF) acknowledges the influence of the child’s environment on development and functioning but in our study, we lacked data pertaining to the household members or the socio-economic environment. The association between the socio-economic status of households and the prevalence of disabilities in CLHIV was not explored and needs additional exploration to better understand the phenomenon.

The researchers assert that children often experiencing functional limitations and disabilities related to other impairments and diseases have not been included in this study. Screening of functional limitations and subsequent follow-up for rehabilitative care are essential for these children and this should be explored further.

## Appendix 1

**TABLE 1-A1:** Child Functioning Scores (*n* = 44).

Questions	Responses of caregivers
**CF1**. I would like to ask you some questions about difficulties your child may have. S (*name*) wear glasses or contact lenses?	1 Yes 3 2 No 41
**CF2**. When wearing his/her glasses or contact lenses, does (*name*) have difficulty seeing? D you say (*name*) has: no difficulty, some difficulty, a lot of difficulty or cannot do at all?	1 No difficulty 3 2 Some difficulty 0 3 A lot of difficulty 0 4 Cannot do at all 0
**CF3**. Does (*name*) have difficulty seeing? D you say (*name*) has: no difficulty, some difficulty, a lot of difficulty or cannot do at all?	1 No difficulty 40 2 Some difficulty 2 3 A lot of difficulty 2 4 Cannot do at all 0
**CF4**. Does (*name*) use a hearing aid?	1 Yes 7 2 No 37
**CF5**. When using his/her hearing aid, does (*name*) have difficulty hearing sounds like peoples’ voices or music? D you say (*name*) has: no difficulty, some difficulty, a lot of difficulty or cannot do at all?	1 No difficulty 0 2 Some difficulty 1 3 A lot of difficulty 6 4 Cannot do at all 0
**CF6**. Does (*name*) have difficulty hearing sounds like peoples’ voices or music? D you say (*name*) has: no difficulty, some difficulty, a lot of difficulty or cannot do at all?	1 No difficulty 38 2 Some difficulty 2 3 A lot of difficulty 4 4 Cannot do at all 0
**CF7**. Does (*name*) use any equipment or receive assistance for walking?	1 Yes 0 2 No 44
**CF8**. Without his/her equipment or assistance, does (*name*) have difficulty walking 100 yards/meters on level ground? That would be about the length of 1 football field. [Or insert country- specific example]. D you say (*name*) has: some difficulty, a lot of difficulty or cannot do at all?	N/A 1 No difficulty 0 2 Some difficulty 0 3 A lot of difficulty 0 4 Cannot do at all 0
**CF9**. Without his/her equipment or assistance, does (*name*) have difficulty walking 500 yards/meters on level ground? That would be about the length of 5 football fields. [Or insert country specific example]. D you say (*name*) has: no difficulty, some difficulty, a lot of difficulty or cannot do at all?	N/A 1 No difficulty 0 2 Some difficulty 0 3 A lot of difficulty 0 4 Cannot do at all 0
**CF10**. With his/her equipment or assistance, does (*name*) have difficulty walking 100 yards/meters on level ground? That would be about the length of 1 football field. [Or insert country-specific example]. D you say (*name*) has: no difficulty, some difficulty, a lot of difficulty or cannot do at all?	N/A 1 No difficulty 0 2 Some difficulty 0 3 A lot of difficulty 0 4 Cannot do at all 0
**CF11**. With his/her equipment or assistance, does (*name*) have difficulty walking 500 yards/meters on level ground? That would be about the length of 5 football fields. [Or insert country specific example]. D you say (*name*) has: no difficulty, some difficulty, a lot of difficulty or cannot do at all?	N/A 1 No difficulty 0 2 Some difficulty 0 3 A lot of difficulty 0 4 Cannot do at all 0
**CF12**. Compared with children of the same age, does (*name*) have difficulty walking 100 yards/meters on level ground? That would be about the length of 1 football field. [Or insert country-specific example]. D you say (*name*) has: no difficulty, some difficulty, a lot of difficulty or cannot do at all?	1 No difficulty 39 2 Some difficulty 5 3 A lot of difficulty 0 4 Cannot do at all 0
**CF13**. Compared with children of the same age, does (*name*) have difficulty walking 500 yards/meters on level ground? That would be about the length of 5 football fields. [Or insert country-specific example]. D you say (*name*) has: no difficulty, some difficulty, a lot of difficulty or cannot do at all?	1 No difficulty 39 2 Some difficulty 5 3 A lot of difficulty 0 4 Cannot do at all 0
**CF14**. Does (*name*) have difficulty with self-care such as feeding or dressing him/herself? D you say (*name*) has: no difficulty, some difficulty, a lot of difficulty or cannot do at all?	1 No difficulty 34 2 Some difficulty 8 3 A lot of difficulty 0 4 Cannot do at all 2
**CF15**. When (*name*) speaks, does he/she have difficulty being understood by people inside of this household? D you say (*name*) has: no difficulty, some difficulty, a lot of difficulty or cannot do at all?	1 No difficulty 36 2 Some difficulty 5 3 A lot of difficulty 1 4 Cannot do at all 2
**CF16**. When (*name*) speaks, does he/she have difficulty being understood by people outside of this household? D you say (*name*) has: no difficulty, some difficulty, a lot of difficulty or cannot do at all?	1 No difficulty 35 2 Some difficulty 6 3 A lot of difficulty 3 4 Cannot do at all 0
**CF17**. Compared with children of the same age, does (*name*) have difficulty learning things? D you say (*name*) has: no difficulty, some difficulty, a lot of difficulty or cannot do at all?	1 No difficulty 28 2 Some difficulty 9 3 A lot of difficulty 6 4 Cannot do at all 1
**CF18**. Compared with children of the same age, does (*name*) have difficulty remembering things? D you say (*name*) has: no difficulty, some difficulty, a lot of difficulty or cannot do at all?	1 No difficulty 28 2 Some difficulty 8 3 A lot of difficulty 6 4 Cannot do at all 2
**CF19**. Does (*name*) have difficulty concentrating on an activity that he/she enjoys doing? D you say (*name*) has: no difficulty, some difficulty, a lot of difficulty or cannot do at all?	1 No difficulty 27 2 Some difficulty 11 3 A lot of difficulty 6 4 Cannot do at all 0
**CF20**. Does (*name*) have difficulty accepting changes in his/her routine? D you say (*name*) has: no difficulty, some difficulty, a lot of difficulty or cannot do at all?	1 No difficulty 21 2 Some difficulty 11 3 A lot of difficulty 11 4 Cannot do at all 1
**CF21**. Compared with children of the same age, does (*name*) have difficulty controlling his/her behaviour? D you say (*name*) has: no difficulty, some difficulty, a lot of difficulty or cannot do at all?	1 No difficulty 21 2 Some difficulty 10 3 A lot of difficulty 13 4 Cannot do at all 0
**CF22**. Does (*name*) have difficulty making friends? D you say (*name*) has: no difficulty, some difficulty, a lot of difficulty or cannot do at all?	1 No difficulty 32 2 Some difficulty 8 3 A lot of difficulty 3 4 Cannot do at all 1
**CF23**. How often does (*name*) seem very anxious, nervous or worried? D you say: daily, weekly, monthly, a few times a year or never?	1 Daily 2 2 Weekly 8 3 Monthly 5 4 A few times a year 5 5 Never 24
**CF24**. How often does (*name*) seem very sad or depressed? D you say: daily, weekly, monthly, a few times a year or never?	1 Daily 1 2 Weekly 9 3 Monthly 4 4 A few times a year 7 5 Never 23
